# A pilot randomized controlled trial comparing the efficacy of exercise, spinal manipulation, and neuro emotional technique for the treatment of pregnancy-related low back pain

**DOI:** 10.1186/2045-709X-20-18

**Published:** 2012-06-13

**Authors:** Caroline D Peterson, Mitchell Haas, W Thomas Gregory

**Affiliations:** 1 Private Practice, Portland, OR, USA; 2 Division of Research, University of Western States, Portland, OR, USA; 3 Department of Obstetrics & Gynecology, Oregon Health & Science University, Portland, OR, USA

## Abstract

**Background:**

This pilot randomized controlled trial evaluated the feasibility of conducting a full scale study and compared the efficacy of exercise, spinal manipulation, and a mind-body therapy called Neuro Emotional Technique for the treatment of pregnancy-related low back pain, a common morbidity of pregnancy.

**Methods:**

Healthy pregnant women with low back pain of insidious onset were eligible to enroll in the study at any point in their pregnancy. Once enrolled, they remained in the study until they had their babies. Women were randomly allocated into one of three treatment groups using opaque envelopes. The treatment schedule paralleled the prenatal care schedule and women received individualized intervention. Our null hypothesis was that spinal manipulation and Neuro Emotional Technique would perform no better than exercise in enhancing function and decreasing pain. Our primary outcome measure was the Roland Morris Disability Questionnaire and our secondary outcome measure was the Numeric Pain Rating Scale. Intention to treat analysis was conducted. For the primary analysis, regression was conducted to compare groups on the outcome measure scores. In a secondary responder analysis, difference in proportions of participants in attaining 30% and 50% improvement were calculated. Feasibility factors for conducting a future larger trial were also evaluated such as recruitment, compliance to study protocols, cost, and adverse events.

**Results:**

Fifty-seven participants were randomized into the exercise (n = 22), spinal manipulation (n = 15), and Neuro Emotional Technique (n = 20) treatment arms. At least 50% of participants in each treatment group experienced clinically meaningful improvement in symptoms for the Roland Morris Disability Questionnaire. At least 50% of the exercise and spinal manipulation participants also experienced clinically meaningful improvement for the Numeric Pain Rating Scale. There were no clinically meaningful or statistically significant differences between groups in any analysis.

**Conclusions:**

This pilot study demonstrated feasibility for recruitment, compliance, safety, and affordability for conducting a larger study in the future. Spinal manipulation and exercise generally performed slightly better than did Neuro Emotional Technique for improving function and decreasing pain, but the study was not powered to detect the between-group differences as statistically significant.

**Trial registration:**

ClinicalTrials.gov (Identifier: NCT00937365).

## Background

Pregnancy-related low back pain (PRLBP) is pain of insidious onset in the lumbar or sacroiliac region that begins during pregnancy [1,2]. Over half of all pregnant women experience PRLBP [3,4] and the natural trajectory of the condition is intensification of pain over the course of pregnancy [2,5]. Low back pain experienced due to pregnancy is poorly understood and conjectured to result from suboptimal biomechanics [6], an increase in relaxin [7], or the evolution to bipedalism [8]. The literature on risk factors for PRLBP is inconsistent. In a summary review of 25 studies of risk factors for PRLBP the only consistent risk factor was a history of lumbar, pubic symphysis, or sacroiliac pain [9].

PRLBP is often (68%) unreported to health care providers [4], despite two thirds of pregnant women requiring sick leave secondary to PRLBP [10]. Of those mothers who report pain, only 25% are given recommendations for symptom management [4]. Over one third of untreated women continue to experience low back pain in the year after pregnancy [4,11,12]; this trend persists up to six years postpartum [13]. Those women who continue to experience low back pain in the postpartum are also more susceptible to concomitant morbidities such as postpartum depression [14]. For these reasons effective management of PRLBP is important to investigate.

Exercise [15], spinal manipulative therapy (SMT) [16,17], and a mind-body therapy called Neuro Emotional Technique (NET) that addresses the emotional component of a condition [18] have been demonstrated to be effective treatments for low back pain in non-pregnant populations. Exercise [19,20] and chiropractic SMT [21,22] have also been demonstrated to decrease PRLBP, however the quality of the chiropractic SMT studies is poor to moderate [23]. One pilot randomized controlled trial of osteopathic SMT for PRLBP was identified [24]. In that study no high velocity low amplitude thrusts were used. The study reported PRLBP did improve in the treatment group, but did not reach statistical significance when compared to the control group. Roland Morris disability scores worsened in all groups, although the control group worsened more than the treatment group. No studies were identified that evaluated NET for the treatment of PRLBP.

Our pilot study responded to the Cochrane Review’s call for more research on the treatment of PRLBP [2]. In this study we evaluated the feasibility of conducting a larger randomized trial with consideration to recruitment, compliance to study protocols, cost, and adverse events. The pilot was also designed to compare the three treatment groups in order to estimate an effect size for disability and pain outcomes to inform the sample size of a future randomized controlled trial.

## Methods

### Design

This pilot parallel group design randomized controlled trial was conducted at Oregon Health & Science University through the Department of Obstetrics & Gynecology between August 2009 and April 2011. A total of 57 participants were enrolled. The trial evaluated the relative efficacy [25] of individualized treatment with exercise (control) (n = 22), SMT (n = 15), or NET (n = 20) for pregnancy-related low back pain. The intervention is discussed in detail below. Our primary null hypothesis was that SMT and NET performed no better than exercise for the treatment of PRLBP as measured by the Roland Morris Disability Questionnaire. Our secondary null hypothesis was that SMT and NET performed no better than exercise for the treatment of PRLBP as measured by an 11-point Numeric Pain Rating Scale.

All participants gave oral and written informed consent and received $20 for each study visit, which took about one hour to complete. The study was approved by Oregon Health & Science University’s Institutional Review Board.

### Study participants

Women were eligible for the study if they were pregnant with a singleton and had low back pain of unknown origin that began during pregnancy and was reproducible by manual palpation. Women with health conditions that contraindicated exercise (heart disease, diabetes, thyroid disease, hypertension, Body Mass Index greater than 40, infection, incompetent cervix, bleeding, ruptured membranes, severe anemia, intrauterine growth restriction, poorly controlled seizure disorder, thrombophlebitis, decreased fetal movement, amniotic fluid leakage) [26] or manipulation (unrelenting fever, unrelenting night pain, loss of bowel or bladder control, progressive neurological deficit, direct trauma, unexplained weight loss, radiating pain below the knee, cancer, spinal fracture or tumors, blood dyscrasias) [27] were excluded from consideration along with women who smoked, consumed alcohol, were medicated with anti-depressants, or had a Roland Morris score above 20 or below 4 [28]. Women were also excluded if they were planning to move during pregnancy, did not read and write English, were not willing to comply with the study visit schedule, or were not willing to be randomized into any of the treatment arms.

Before being randomized participants identified their treatment preference (*i.e.* exercise, SMT, NET, or none). Treatment preference is a well known predictor of outcomes and thus is a potential confounding variable [29-31]. The randomization schedule was completed prior to initiating the study and was concealed from all study staff by using consecutively numbered, sealed, opaque envelopes for each strata of preference group. Once a participant was found to be eligible, she would open the consecutive envelope in her preference strata in the presence of the researcher. Participants and the practitioner providing the intervention were not blinded to the intervention after randomization.

### Study protocol

Study participants were recruited by fliers in prenatal clinics and by community, magazine, and internet announcements in the Portland, Oregon metropolitan area. Potential participants were first screened by phone and if study eligibility requirements were met, the initial study visit was scheduled. At the initial screening and prior to randomization each treatment group was described so all participants were familiar with all treatments. Participants could enter the study at any point in their pregnancy and remained in the study until they had their baby. All participants continued to receive their usual obstetric or midwifery prenatal care following randomization. The RCT treatment schedule paralleled the prenatal care schedule (once monthly until 28 weeks gestation, twice monthly until 36 weeks gestation, and weekly thereafter).

When the participant arrived for each study treatment visit she completed the primary and secondary outcome measures, drew a pain diagram, and responded to questions about adverse events, treatment by other practitioners, new injuries, and use of medication. After filling out the paperwork all participants were palpated and assessed with standard orthopedic and neurologic exams to ensure they were safe to treat. Each was also palpated to determine if intersegmental dysfunctions were present. Following the assessment each participant was treated in accordance with the protocol for their treatment arm, which is described below.

### Intervention

The lead author served as the therapist and has practiced chiropractic medicine for 15 years. At the baseline visit she screened volunteers for study eligibility through case history and standard orthopedic and neurologic tests. Participants in all three treatment groups were advised to use ice after each treatment since a little soreness is commonly experienced after treatment.

### Exercise

Exercise participants were given a booklet and enthusiastically instructed on pelvic tilts, pelvic floor, gluteus maximus, latissimus dorsi, and hip adductor strengthening exercises to promote low back stability and flexibility. The specific exercises in the booklet have been shown to decrease PRLBP [32]. The booklet also instructed exercise participants on recommendations for postural and movement patterns that help alleviate low back pain. Finally, warnings in the booklet about when to stop exercising were reviewed with the participant. At each study visit exercises and lifestyle suggestions were reviewed and practiced with participants. Additional individualized stretching or strengthening exercises were prescribed, demonstrated, and practiced at each study visit based on muscle strength and flexibility assessment. Exercises took about 15 min to perform at home and participants were requested to exercise five times weekly.

### Spinal Manipulative Therapy

Participants in the SMT group were palpated to determine if each had intersegmental dysfunction prior to manipulating [33]. Hypomobile joints were isolated through positioning, then a slow force was applied to preload the joint at the physiological end range. After loading the joint, a high velocity, low amplitude thrust was applied to the isolated joint to move it just past the physiological end range in the side posture position for lumbar and sacroiliac lesions. The thrust was applied in the direction, velocity, and amplitude as determined by the clinician from the palpation exam findings [33]. A hypermobile joint or region was stabilized by creating a fulcrum at a specific joint by the participant lying on padded blocks [34]. The blocks were always used to adjust a Sacro Occipital Technique Category II pelvis, and the Activator was always used to adjust the pubic symphysis [35].

### Neuro emotional technique

Neuro Emotional Technique (NET) is a chiropractic mind-body technique that combines desensitization procedures (such as relaxed breathing and visualization) with elements of Five Element Chinese medicine (such as the association of emotions with certain organs or meridians) and chiropractic medicine (the adjustment of the spinal levels that innervate the organ in question) in an attempt to address cognitive distortions through the use of a semantic algorithm [36]. The reliability and validity of the manual muscle testing used to guide the technique are unknown [37], but muscle testing is considered to be part of the treatment package of NET. The NET standard protocol was followed [38].

### Outcomes

At each assessment visit, each participant completed a form indicating if she was following the study protocol, was taking any pain medication, had been injured outside of the study, or had experienced any adverse effects as a result of the intervention. Participants were asked to rate their low back function and pain “today” as measured by the Roland Morris Disability Questionnaire (primary outcome) and an 11-point Numeric Pain Rating Scale (secondary outcome). The Roland Morris Disability Questionnaire is a 24-item index of activities of daily living related to low back function that has been shown to be valid, reliable, and sensitive for measuring changes in mild to moderate disability [39,40]. The 11-point Numeric Pain Rating Scale (0–10) has been shown to be valid [41]. A four point change on the Roland Morris Disability Questionnaire, a two point change on the Numeric Pain Rating Scale, or a 30% change in either from the baseline is considered to be clinically meaningful within-persons [41-44].

### Statistical analysis

Intention-to-treat analysis was conducted with participants included in the original assigned group. In the primary analysis, linear regression was used to compare the three groups with follow-up disability and pain scores as continuous dependent variables. The covariates included in all between group models were gestational age at entrance into the study, baseline score of the outcome measure, maternal age, and history of low back pain. The endpoint was the eighth study visit assessment or the last study visit if the participant did not complete eight visits.

A secondary responder analysis was conducted for the Roland Morris Disability Questionnaire and Numeric Pain Rating Scale. Two categorical outcome variables were created to reflect a minimum of 50% improvement (yes/no), and a minimum of 30% improvement (yes/no). Differences in the proportion of responders between groups were then calculated. Thirty percent improvement is considered to be the minimal change needed to identify clinically meaningful improvement [44,45]. However, 50% improvement is typically the standard used in responder analysis [46,47].

Missing data were imputed using the last observation carried forward [48,49]. Although last observation carried forward method of controlling for missingness is biased toward participants not worsening over the remaining segment of the study, we elected to use this method so our results could be compared with a similarly designed osteopathic trial of treatment for pregnancy related low back pain [24]. To determine if this approach was appropriate for our data, we also conducted a sensitivity analysis excluding participants with only baseline data.

Statistical significance was set at 0.05 for all tests with no correction for multiple group comparisons. Sample size was limited by available funds and the enrollment window established for this pilot; it was not determined by *a priori* power analysis. A post hoc power analysis was conducted to identify what effect sizes were detectable with 80% power at the 0.05 level of significance. A study is said to be adequately powered if the detectable effect size is no larger than a clinically important difference between groups. Statistical analysis was performed with SPSS v18.0 (SPSS, Inc, Chicago, IL).

## Results

**Figure 1:**
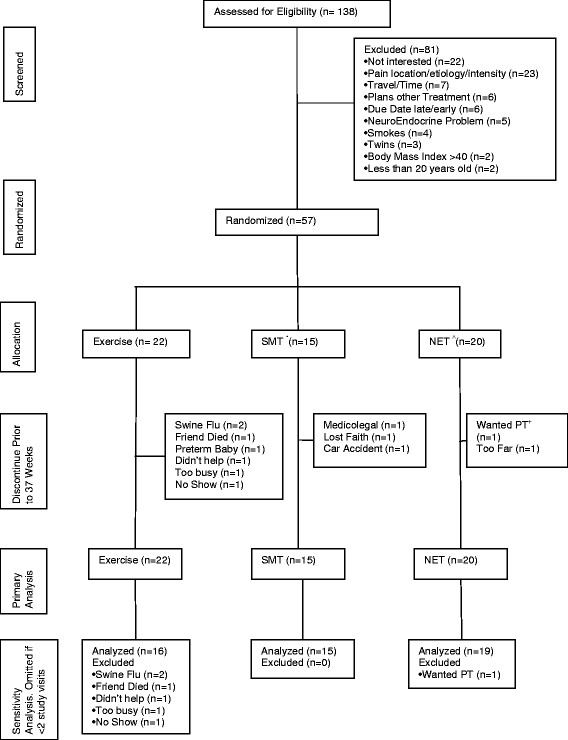
Flow of Participants Through the Trial.

We screened 138 pregnant women (approximately 10 per month) for eligibility and 57 were randomized (Figure 1). Twenty-two participants were randomized into the exercise group, 20 were randomized into the NET group, and 15 were randomized into the SMT group. Six exercise participants and one NET participant discontinued the study after the first visit for reasons listed in Figure 1.

### Participant baseline characteristics

Baseline health characteristics and demographics were somewhat balanced across groups (Tables 1, 2 and 3). Women in the exercise group were less likely to be married, to work during pregnancy, and to have good or quite good self-perceived general health status prior to pregnancy. Additionally, their low back pain began earlier in pregnancy and they were more likely to be taking medication for pain, and to have missed work because of the pain than were the women in the other treatment groups. Finally, women in the exercise group also entered the study earlier in their pregnancies than those in the NET or SMT groups, and perhaps because of this had a lower BMI at their first study visit. If the seven women who withdrew from the study immediately upon randomization were not included in the descriptive analysis, the study groups were balanced across all variables except women in the exercise group were less likely to have good or quite good health prior to pregnancy.

**Table 1:** Baseline demographics of participants by treatment group and total

**Variable**	**Exercise (n = 22)**	**NET (n = 20)**	**SMT (n = 15)**	**Total (n = 57)**
	**Mean (SD) or n (%)**	**Mean (SD) or n (%)**	**Mean (SD) or n (%)**	**Mean (SD) or n (%)**
Age, years	28.7 (5.1)	29.7 (5.5)	31.1 (4.2)	29.7 (5.0)
Race/ethnicity				
White	17 (77.3%)	17 (65.0%)	10 (66.7%)	44 (77.2%)
Black	0	0	2 (13.3%)	2 (3.5%)
Asian	1 (4.5%)	0	1 (6.7%)	2 (3.5%)
Hispanic	4 (18.2%)	3 (15.0%)	2 (13.3%)	9 (15.8%)
Education				
High School or Less	5 (22.7%)	2 (10.0%)	2 (13.3%)	9 (16.3%)
Some College or Tech/Trade School	7 (31.8%)	9 (45.0%)	5 (33.3%)	21 (36.8%)
4 Year College	6 (27.3%)	2 (10.0%)	6 (40.0%)	14 (24.6%)
Graduate School	4 (18.2%)	7 (35.0%)	2 (7.4%)	13 (22.8%)
Married	13 (59.1%)	17 (85.0%)	11 (73.3%)	41 (71.9%)
Work During Pregnancy	15 (68.2%)	17 (85.0%)	12 (80.0%)	44 (77.2%)

^*^SMT = Spinal Manipulative Therapy, ^^^NET = Neuro Emotional Technique, ^+^PT = Physical Therapy.

**Table 2:** Baseline health characteristics of participants by treatment group and total

**Variable**	**Exercise (n = 22)**	**NET (n = 20)**	**SMT (n = 15)**	**Total (n = 57)**
	**Mean (SD) or n (%)**	**Mean (SD) or n (%)**	**Mean (SD) or n (%)**	**Mean (SD) or n (%)**
Gravida	3* (1.8)	2* (2.3)	3* (2.7)	3* (1.9)
Para	0* (1.2)	1* (1.3)	1* (0.9)	1* (1.1)
Body Mass Index at study start	26.9 (3.9)	28.2 (4.4)	29.3 (4.4)	27.9 (4.3)
Took Medication for Pain at Study Start	4 (18.2%)	2 (10.0%)	1 (6.7%)	7 (12.3%)
Very Good or Quite Good Health Prior to Pregnancy	18 (81.8%)	20 (100.0%)	15 (100.0%)	53 (93%)
Exercised Prior to Pregnancy				
Daily	4 (18.2%)	5 (25.0%)	2 (13.3%)	11 (19.3%)
Weekly	9 (40.9%)	8 (40.0%)	7 (46.7%)	24 (42.1%)
Occasional	6 (27.3%)	6 (30.0%)	6 (40.0%)	18 (31.6%)
Never	3 (13.6%)	1 (5.0%)	0	4 (7.0%)
History of low back pain	10 (45%)	13 (65%)	8 (53%)	31 (54%)
Gestational age at onset of this episode	11.7 (6.1)	13.9 (6.6)	16.1 (5.8)	25.4 (6.4)
Sick Leave due to PRLBP	5 (33%)	4 (24%)	2 (17%)	11 (25%)
Pain Location				
Lumbosacral	10 (45.5%)	12 (60.0%)	7 (46.7%)	29 (50.9%)
Pelvis	6 (27.3%)	4 (20.0%)	2 (13.3%)	12 (21.0%)
Lumbar	3 (13.6%)	3 (15.0%)	2 (13.3%)	8 (14.0%)
Composite	3 (13.6%)	1 (5.0%)	2 (13.3%)	6 (10.5%)

*Median.

**Table 3:** Treatment and gestational age

**Variable**	**Exercise (n = 22)**	**NET (n = 20)**	**SMT (n = 15)**	**Total (n = 57)**
	**Mean (SD) or n (%)**	**Mean (SD) or n (%)**	**Mean (SD) or n (%)**	**Mean (SD) or n (%)**
Gestational age				
Baseline	23.7 (7.5)	27.0 (5.8)	25.7 (5.3)	25.4 (6.4)
Follow-up*	33.0 (8.0)	35.9 (4.8)	36.2 (4.5)	34.8 (6.3)
Treatment Preference at baseline.				
No Preference	6 (27.3%)	5 (25.0%)	4 (26.7%)	15 (26.3%)
Exercise	2 (9.1%)	2 (10.0%)	2 (13.3%)	6 (10.5%)
NET	7 (31.8%)	6 (30.0%)	3 (20.0%)	16 (28.1%)
SMT	7 (31.8%)	7 (35.0%)	6 (40.0%)	20 (35.1%)
Total number of treatments	5.4 (0.7%)	7.8 (0.9%)	7.5 (0.6%)	6.8 (3.5%)

* Gestational Age at the last visit (for which outcomes were used in the analysis).

### Outcomes data

The majority in all three groups had clinically meaningful improvement in function (4 point or 30% improvement on the Roland-Morris), and the majority of participants in the exercise and SMT groups also had clinically meaningful improvement in pain (2 point or 30% improvement on the numeric rating scale) (Tables 4 &5). The SMT group attained a minimal clinical important improvement in 80% of the participants for function and 67% for pain. Smaller proportions were observed in the other two groups.

**Table 4:** Roland morris disability questionnaire within-group continuous and categorical comparisons for pre- and post-intervention

	**Continuous data**		**Categorical data**		
	**Baseline**	**Endpoint**	**4+ Point Improve**	**30% Improve**	**50% Improve**
	**Mean (SD)**	**Mean (SD)**			
Exercise (n = 22)	10.7 (4.9)	6.1 (5.9)	60%	68%	55%
NET (n = 20)	9.3 (3.7)	5.7 (4.7)	60%	60%	50%
SMT (n = 15)	8.7 (4.1)	4.1 (4.3)	50%	80%	67%

* Significance of raw score change using a repeated-measures *t*-test.

**Table 5:** Numeric pain rating scale within-group continuous and categorical comparisons for pre- and post-intervention

	**Continuous data**		**Categorical data**		
	**Baseline**	**Endpoint**	**2+ Point improve**	**30% Improve**	**50% Improve**
	**Mean (SD)**	**Mean (SD)**			
Exercise (n = 22)	3.9 (1.5)	2.4 (1.8)	55%	64%	55%
NET (n = 20)	3.2 (1.4)	2.4 (1.6)	35%	45%	35%
SMT (n = 15)	3.5 (1.1)	1.9 (1.7)	60%	67%	53%

* Significance of raw score change using a repeated-measures *t*-test.

### Between-groups comparisons

No statistically significant differences between groups were noted in either the Roland Morris Disability Questionnaire or Numeric Pain Rating Scale scores in the pair-wise comparison of treatment groups in either the primary or secondary analyses (Tables 6 &7). All confidence intervals were wide for the between-groups comparisons, demonstrating imprecision in the treatment effect point estimates and lack of power to detect differences between groups.

**Table 6:** Roland morris disability questionnaire between-group comparisons

**Comparison groups**	**Outcome score**			**30% Improvement**					**50% Improvement**						
	**Crude mean difference (95% CI)**	**Adjusted mean difference^1^ (95% CI)**	**P**	**Crude proportional difference (95% CI)**	**P**	**Crude proportional difference (95% CI)**	**P**								
Ex^2^ vs NET	−0.3 (−3.7, 3.0)	0.7 (−2.9, 4.2)	.712	0.08 (−0.21, 0.37)	.530	0.05 (−0.26, 0.36)	.760
Ex^2^ vs SMT	−2.0 (−5.6, 1.6)	0.01 (−3.2, 3.2)	.995	−0.12 (−0.41, 0.17)	.420	−0.12 (−0,45, 0.21)	.239
SMT^2^ vs NET	1.6 (−1.5, 4.8)	1.2 (−2.1, 4.5)	.453	0.2 (−0.13, 0.53)	.881	0.18 (−0.06, 0.42)	.841

^1^ Adjusted for Gestational Age at entrance into the study, Outcome Measure score at baseline, Maternal age, and history of low back pain using linear regression for testing adjusted mean differences of outcome scores.

^2^ Reference Category: A positive mean difference favors the reference category.

**Table 7:** Numeric pain rating scale between-group comparisons

**Comparison groups**	**Outcome score**			**30% Improvement**		**50% Improvement**	
	**Crude mean difference (95% CI)**	**Adjusted mean difference^1^ (95% CI)**	P	**Crude proportional difference (95% CI)**	**P**	**Crude proportional difference (95% CI)**	**P**
Ex^2^ vs NET	−0.1 (−1.1, 1.0)	0.1 (−1.0, 1.3)	.818	0.19 (−0.12, 0.50)	.898	0.20 (−0.11, 0.51	.894
Ex^2^ vs SMT	−0.5 (−1.8, 0.7)	−0.3 (−1.5, 1.0)	.656	−0.03 (−0.34, 0.28)	.425	0.02 (−0.29, 0.33)	.552
SMT^2^ vs NET	0.5 (−0.7, 1.6)	0.5 (−0.8, 1.7)	.442	0.22 (−0.09, 0.53)	.916	0.18 (−0.13, 0.49)	.871

^1^ Adjusted for Gestational Age at entrance into the study, Outcome Measure score at baseline, Maternal age, and history of low back pain using linear regression for testing adjusted mean differences of outcome scores^2^ Reference Category: A positive mean difference favors the reference category.

### Sensitivity analysis

The results of the sensitivity analysis were consistent with the primary analysis when the seven individuals with only baseline data were excluded. One comparison (Exercise *vs.* NET) became statistically significant for the Numeric Pain Rating Scale. The primary analysis was the most conservative analysis performed. This suggests that our method of imputing missing data using last observation carried forward did not bias our results toward the group with more dropouts, as is sometimes the case.

### Post hoc power analysis

The study had 80% power to detect mean between-group differences of approximately 4.7 points on the Roland-Morris and 1.8 points on the Numeric Pain Rating Scale at the .05 level of significance. The study was not powered to detect a minimal clinically important differences between groups [50,51].

### Feasibility evaluation

#### Adherence to care

Three of the 16 exercise participants who had more than one study visit did not follow the prescribed study visit schedule. Because study participants could enter the study at any point in their pregnancy, 5 (23%) exercise participants, 6 (30%) NET participants, and 3 (20%) SMT participants completed eight study visits. Seven (38%) of the exercise participants who had more than one study visit reported that they did not perform their exercises at least five times a week. However, all seven reported between 40% - 100% improvement in function. Five of the seven reported 50%-75% improvement in pain intensity; pain intensity did not change for one participant and worsened for another who did not perform her exercises regularly.

#### Study costs

The study cost for recruitment, participation, software, and supplies was approximately $210 per participant. A subsample also participated in more extensive data collection and a three month postpartum follow-up that will be reported in a future article. The subsample’s additional expense for participation, lab work, and maternal-infant attachment assessment were approximately $250 for each person.

#### Care outside the study

Only two NET study participants reported accessing care for their low back pain outside of the study. One of these NET participants did so after she injured her back lifting a box and required care while the study clinician was out of town. A third exercise participant failed to report whether she had sought care for her low back pain outside of the study at one of the study visits.

#### Adverse outcomes

No adverse events were reported by study participants in any group. However, 6% of exercise and SMT study visits produced soreness, while 18% of NET study visits produced soreness.

#### Participant feedback

Study participants were asked for feedback about the study when they reached 37 weeks gestation. All women reported enjoying participating in the study. All but one mother surveyed reported satisfaction with the improvement she experienced in symptoms or in stabilization of symptoms. Because pregnancy-related low back pain worsens over the course of pregnancy, women were satisfied with their outcome even if symptoms did not improve because they compared their pain and disability for this pregnancy with those from a previous pregnancy.

I found the study very helpful in getting rid of a large amount of pain I have been experiencing since the beginning of my pregnancy. The exercise allowed me to do activities that otherwise would have been too painful to participate in, and I believe it has helped prepare my body for labor and after the baby arrives (EX19).

I noticed a dramatic decrease in the amount and intensity of the low back pain I experienced. I also was able to become aware of some issues regarding this pregnancy and my past that I wasn’t aware of consciously. That was helpful because it made me make more of an effort to bond with this baby when sometimes I would feel distracted (*i.e.* when work/family responsibilities would take over) (NET05).

I think the study was really good for me. I feel much better, the pain decreased a lot. I also think that my baby liked it. I really enjoy being on this study and I definitely recommend it for all pregnant women. It also helped me to feel more relaxed (SMT05).

## Discussion

This was the first identified RCT to compare the relative efficacy of three conservative interventions for the treatment of PRLBP and to show improvement in pain and function with care. The function and pain improvement within the SMT and exercise groups were clinically meaningful (dependent *t*-test p < 0.002)). This is important because the only other RCT identified to use manual medicine intervention for the treatment of PRLBP was an osteopathic pilot study that found Roland Morris Disability scores worsened over time in all groups, but function deteriorated less in the osteopathic manipulation group than in the placebo or control group. They also found pain intensity improved by less than half a point on a 11-point pain scale in the osteopathic manipulation group, and there was no statistically significant difference from the placebo or control groups [24]. In the osteopathic pilot RCT [24], our pilot RCT, and other studies [4] PRLBP was mild to moderate in severity and provoked mild to moderate disability. Participants in our study had Roland Morris scores that were approximately 2 points worse at study entrance than those in the osteopathic study. Our participants experienced Numeric Pain Rating Scale scores that were about 1 point less intense than in the osteopathic study. In contrast to the osteopathic PRLBP pilot our participants experienced more improvement in their Roland Morris scores than in their pain scores. This could be due to a floor effect for the pain score since our baseline pain measures were lower than in the osteopathic study.

We have demonstrated the feasibility of recruiting a sufficient sample in a reasonable time for a future full-scale RCT and that there is reasonable adherence to treatment and compliance with follow-up. We found that only 11% of our study participants preferred to be randomized into the exercise group while 28% preferred NET and 35% preferred SMT. This could explain, in part, why we had 27% attrition from the exercise group immediately following randomization while only one participant (5%) in the NET arm withdrew immediately following randomization, and no SMT participants withdrew immediately following randomization. Exercise appears to appeal to only a sub-group of women. Thus, because all treatments were associated with some improvement, a future large randomized controlled trial for the conservative treatment of pregnancy-related low back pain might consider comparing SMT, which performed better than other treatment interventions and was preferred by participants, to a standard of care control group so the number of participants in each study arm would be reasonable to recruit and retain.

It is important to further investigate conservative intervention options such as manual medicine, exercise, and mind-body therapy for the treatment of PRLBP, since pharmaceutical management of pain during pregnancy has been poorly researched and the short- and long-term consequences of pharmaceutical management during pregnancy are largely unknown for the mother and the fetus [52]. Additionally, passive intervention for the treatment of PRLBP using support belts is not well substantiated by the literature [53]. In non-pregnant populations the use of support belts promotes deconditioning and the possibility of injury [54]. The potential safety of exercise, SMT, and NET for a pregnant population with low back pain was demonstrated in this pilot study and paralleled the safety demonstrated for SMT in non-pregnant populations [17,23,55-58]. If safety and effectiveness can be demonstrated in a larger study of conservative intervention for PRLBP, chiropractic care for PRLPB might be a valuable option since chiropractic low back pain management is approximately 60% more cost effective in a non-pregnant population than traditional biomedical care after controlling for case complexity [59]. Finally, although this pilot study was not powered for generalizability, it did substantiate other reports that exercise and manual medicine care for PRLBP are beneficial with low associated risk [21,23,60].

Another reason it is important to further investigate the treatment of PRLBP is because it appears to signify an underlying weakness in the musculoskeletal system that, if not addressed during pregnancy, portends future suffering. Women who experience PRLBP and are not treated during pregnancy are at increased risk of experiencing back pain at 3, 6, and 12 months postpartum, and at 3 and 6 years postpartum when compared with women who did not have back pain during pregnancy and women who had back pain during pregnancy and received treatment [13,61,62]. Treatment of PRLBP might not only alleviate low back pain during pregnancy, but could prevent future episodes of low back pain thus saving the health care system unnecessary expenditures [63]. Pregnancy is widely conceptualized as a testing ground for unmasking weaknesses in organ systems that would not appear until later life but for pregnancy [64]. Pregnancy disorders predicting later related health problems have been best established for gestational diabetes predicting diabetes type II [65] and for preeclampsia predicting chronic hypertension [66]. Biomechanical evidence suggests that the female lumbar spine is well designed to respond to fetal load [8], thus PRLBP is not necessarily an inherent morbidity of pregnancy, but might be another warning sign of future ill health just as are gestational diabetes and preeclampsia.

### Limitations and future directions

This feasibility study was designed to inform the construction of a future sufficiently powered RCT of PRLBP. Several limitations flawed this study. One major limitation was that the first author conducted all aspects of the trail including provision of care to all study participants. As a result, she was not blinded to intervention during the analysis phase, insufficient time was available for satisfactory recruiting, and treatment effects could be related to investigator proclivities rather than to the modalities themselves. In a future well funded study separate individuals will randomize participants, treat participants, and analyze the data. Also, more active engagement of prenatal care practitioners can be ensured to enhance recruitment by building on the relationships that were formed during this study.

Another limitation was that allowing women to enter the study at any point in their pregnancy made it difficult for latecomers to complete the treatment protocols before birth and also complicated the analysis. We suggest the next trial should be designed so women enter the study at approximately the same point in pregnancy (*e.g.* 28–32 weeks) and complete the eight study visits by 37 weeks gestation. Toward the beginning of the study we also had difficulty retaining the first women who were randomized into the exercise treatment arm. By mid-study we had resolved that issue by strenuous vetting of potential participants to be certain they were willing to participate in the exercise arm of the study.

Finally, the imputation method of last observation carried forward could bias the outcome in favor of the treatment group that had more people who withdrew early in the study or for the group with more women who arrived at their 8^th^ study visit earlier in pregnancy. This is so because low back pain tends to intensify throughout pregnancy. However, we found that the results for the exercise group and NET group were worse when the seven participants who only had baseline measures were included. This suggests imputation by last observation carried forward was a conservative means of inferring outcome and did not prejudicially preference the group with early withdrawals in our study.

## Conclusion

This pilot RCT demonstrated that a larger study is feasible in terms of recruitment in a reasonable timeframe, adequate adherence to care, compliance with follow-up, and safety. All three interventions appear to provide clinically meaningful improvement in function and pain intensity. However, although no between-groups differences were noted in function and pain intensity following treatment, exercise and SMT might have larger treatment effect sizes than does NET. All three interventions merit further investigation.

## Competing interests

This study was funded in part by The ONE Foundation, the research division of the Neuro Emotional Technique. The ONE Foundation did not contribute to the study in any other way. The authors declare that they have no competing interests.

## Authors’ contributions

CDP was responsible for all aspects of the study including study design, recruitment, randomization, treatment, analysis and interpretation of findings, and manuscript preparation. MH was involved in study design, logistics, interpretation of findings, and manuscript preparation. WTG was involved in study design, acquisition of funding, logistics, and manuscript preparation. All authors read and approved the final manuscript.
